# CLRM-08. TRIAL WORKING GROUPS FOR PAEDIATRIC BRAIN TUMOURS

**DOI:** 10.1093/noajnl/vdab112.007

**Published:** 2021-09-21

**Authors:** Ruman Rahman, David Walker, Emma Campbell, Kristian Aquilina

**Affiliations:** 1 University of Nottingham, Nottingham, UK; 2 Great Ormond Street Hospital, London, UK

## Abstract

**INTRODUCTION:**

Brain tumours are the biggest cancer killer in children and young adults. Several recent developments have the potential to change the outlook for these children, including intra-CSF chemotherapy, ultrasound-mediated blood-brain barrier disruption, convection enhanced delivery, polymer delivery systems, electric field therapy, and intra-arterial and intra-nasal chemotherapy. To date, there have been very few clinical trials to evaluate these. In addition, custom-built hardware, novel surgical procedures and the testing and licensing of implantable devices add difficulty at the regulatory level.

**METHODS:**

The authors participated in an international workshop funded by the charity Children with Cancer UK in 2016, where different experimental techniques aimed at optimising CNS drug delivery were discussed. Following this and two subsequent workshops run by the CBTDDC (Children’s Brain Tumour Drug Delivery Consortium), the CBTDDC and the ITCC (Innovative Therapies for Children with Cancer) brain tumour group launched the ‘Clinical Trials Working Group for Central Nervous System Drug Delivery’. This aims to accelerate clinical trials to assess the safety and effectiveness of drug delivery devices for the treatment of paediatric brain tumours.

**RESULTS:**

On 1 March, 2021, CBTDDC and Mr Kristian Aquilina (Consultant Paediatric Neurosurgeon at Great Ormond Street Hospital) hosted the first steering group meeting, comprising 38 leading brain tumour research scientists and clinicians from the UK, EU and US.

**CONCLUSION:**

The ideas generated during the March meeting are driving the agenda for a Clinical Trials Workshop that will be held in the autumn of 2021. In particular, there was agreed consensus that a ‘Roadmap’ document for pre-clinical to clinical translation needs to be created and shared with the paediatric neuro-oncology research community. We present this abstract to the CNS Clinical Trials Meeting to raise awareness of this initiative with the large number of relevant stakeholders who will be attending the event.

